# Advance care planning in critically ill haematology patients

**DOI:** 10.1186/cc13220

**Published:** 2014-03-17

**Authors:** V Metaxa, J Lambert, A Barrow, J De Vos

**Affiliations:** 1 king's College Hospital, London, UK; 2 Royal London Hospital, London, UK; 3 John Radcliffe Hospital, Oxford, UK; 4 Royal Surrey County Hospital, Guildford, UK

## Introduction

Significant improvements in chemotherapy, haematopoietic stem cell transplantation and general intensive care mean that more patients are now living longer and often present to the ICU at various stages of their disease [1,2]. Encouraging patients with haematological malignancy (HM) to express their wishes and values on end of life (EoL) prior to ICU admission would ensure that their autonomy is respected. The aim of our study was to determine whether patients with hM that were admitted and died in the ICU had any form of advance care planning (ACP) documented prior to their admission.

## Methods

Data were collected on all adult patients with HM that were admitted and died in the ICU of a tertiary haematology referral centre in London during the period of 1 year.

## Results

Information was collected for 34 patients and their characteristics are shown in Table 1. In 62% of the patients EoL decisions were made, and do-not-attempt-resuscitation documentation was found in 65% of the cases. Documentation on information exchange, and participation in the deliberation or the decision-making process was found in only 30% of the patients, even though more than 75% of the haematology physicians estimated the prognosis of these patients as moderate (17%) or poor (59%). In the vast majority of cases (31/34) the deaths occurred after withholding or withdrawal of treatment in ICU.

**Table 1:** Patient characteristics

Mean age	30
Median APACHE II score	26
>3 organ support	60%
Leukaemias	<50%

## Conclusion

Only a small number of patients with HM that die in the ICU have documented ACP prior to admission. Since the majority of ICU patients lack personal decision-making capacity, ACP would ensure that care is consistent with patients' wishes, EoL actions are congruent with their values, the burden on family and healthcare providers is alleviated and cost is decreased.
